# Cardiovascular Risk in People Living with HIV: A Preliminary Case Study from Romania

**DOI:** 10.3390/medicina61081468

**Published:** 2025-08-15

**Authors:** Manuela Arbune, Alina Plesea-Condratovici, Anca-Adriana Arbune, Geanina Andronache, Catalin Plesea-Condratovici, Cristian Gutu

**Affiliations:** 1 Clinical Medical Department, “Dunarea de Jos” University, 800008 Galati, Romania; manuela.arbune@ugal.ro (M.A.); cristian.gutu@ugal.ro (C.G.); 2 Clinical Hospital for Infectious Diseases “Sf. Cuv. Parascheva”, 800179 Galati, Romania; 3 Medical Department, “Dunarea de Jos” University, 800008 Galati, Romania; 4 Neurology Department, Fundeni Clinical Institute, 022328 Bucharest, Romania; anca.arbune@icfundeni.ro; 5 Multidisciplinary Integrated Center of Dermatological Interface Research (MIC-DIR), “Dunarea de Jos” University, 800008 Galati, Romania; 6 Public Health Directorate of Galați County, 800055 Galati, Romania; dumitrachegeanina51@yahoo.com; 7 Morphological and Functional Sciences Department, “Dunarea de Jos” University, 800008 Galati, Romania; catalin.plesea@ugal.ro; 8 ”Aristide Serfioti” Military Hospital, 800179 Galati, Romania

**Keywords:** cardiovascular risk, hypertension, dyslipidaemia, non-AIDS-associated comorbidities, HIV/AIDS

## Abstract

*Background and Objectives:* AIDS-related mortality has significantly decreased due to antiretroviral therapy (ART), leading to a substantial increase in average lifespan. Consequently, cardiovascular diseases have become a growing concern among people living with HIV (PLWH). This study aimed to assess the cardiovascular risk profile of people living with HIV receiving ART and to explore the association between traditional and HIV-related factors with increased cardiovascular risk. *Materials and Methods:* We conducted a case study involving 112 PLWH receiving ART at a specialized clinic in southeastern Romania to estimate cardiovascular risk (CVR) using the Data Collection on Adverse Events of Anti-HIV Drugs (D:A:D^®^) score. For participants aged 40 and above, the SCORE2 algorithm was additionally applied. *Results:* Most participants were male and under 40 years of age, including 34 individuals from Romania’s distinct pediatric HIV cohort. We observed a substantial cardiovascular risk burden: abdominal obesity was present in 24.1% of participants, active smoking was reported by 55.4%, and over 70% had low physical activity levels. Among participants aged 40 and above, the D:A:D^®^ and SCORE2 scores were strongly correlated, with an average cardiovascular age exceeding chronological age by a mean of 7.5 years. Although CVR remained similarly low among subgroups of PLWH under 40, the prevalence of metabolic syndrome was higher in patients from the pediatric cohort compared to those diagnosed later. Traditional risk factors—such as age, obesity, hypertension, dyslipidemia, smoking, and alcohol use—as well as elevated C-reactive protein levels, were significantly associated with increased CVR. *Conclusions:* Residual inflammation in PLWH, despite complete viral suppression in combination with metabolic syndrome, is associated with increased cardiovascular risk even in younger and clinically stable populations. Routine integration of metabolic and cardiovascular risk screening into HIV care may support timely prevention and personalized management strategies starting at an early age.

## 1. Introduction

Human immunodeficiency virus (HIV) infection remains a global public health concern. According to the updated data in the Joint United Nations Programme on HIV/AIDS (UNAIDS) report, an estimated 39.9 million people are living with HIV, of whom 30.7 million have access to antiretroviral therapy (ART). This has contributed to a 69% decrease in AIDS-related deaths since 2004 [1].

Despite these advances, mortality among people living with HIV (PLWH) remains higher than that of the general population. As the PLWH population ages, the incidence of non-AIDS co-morbidities increases, particularly cardiovascular disease (CVD) and metabolic complications [2,3]. PLWH exhibit a two- to threefold higher risk of coronary atherosclerosis compared to HIV-negative individuals, even after adjusting for traditional risk factors [4]. Metabolic syndrome (MetS), a key driver of cardiovascular morbidity, affects over 25% of PLWH, especially those receiving ART [5].

The European Association of Preventive Cardiology (EAPC) classifies Romania as a high-risk country for cardiovascular disease according to the SCORE2 model [6]. The World Health Organization (WHO) indicates a prevalence of obesity at 38.2% among the adult population in Romania, which is the highest rate in Europe [7]. This trend, combined with ART-related metabolic changes, increases CVD risk among PLWH in the country [8,9,10]. Currently, over 18,000 people are living with HIV in Romania. However, there is no published data available regarding their cardiovascular or obesity-related risk profiles [11].

The objective of this study is to evaluate cardiovascular risk among PLWH receiving antiretroviral treatment at a specialized clinic in southeastern Romania. The study aimed to identify priority areas for intervention and guide strategies to improve the integration of cardiometabolic risk management into routine HIV care.

## 2. Materials and Methods

### 2.1. Study Design and Setting

We conducted a case study on the health status of PLWH receiving ART and monitored at the HIV/AIDS Day Clinic of the Clinical Hospital for Infectious Diseases in Galați, located in southeastern Romania. HIV/AIDS monitoring and treatment visits were conducted in accordance with local protocol aligned with the recommendations of the European AIDS Clinical Society (EACS) guidelines [12].

All participants provided written informed consent prior to enrollment. The study was conducted in accordance with the Declaration of Helsinki and approved by the institutional ethics committee.

### 2.2. Recruitment of Participants

Of 401 patients actively registered at the clinic (defined as having attended at least one follow-up visit in the last six months), 189 patients were scheduled for their biannual health evaluation in October 2024. After 14 patients missed their visit, 175 were assessed for eligibility and invited to participate.

### 2.3. Inclusion and Exclusion Criteria

**Figure A1:**
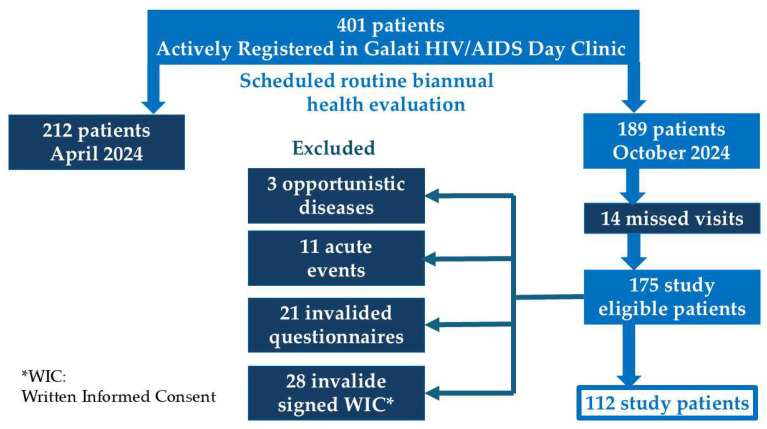
Flow diagram of study patient selection.

The inclusion criteria were the confirmation of HIV infection based on three positive tests (two ELISA and one Western blot), age over 18 years, and a minimum of one year on the current ART regimen. All patients attending the scheduled visit were found eligible for inclusion. Exclusion criteria were as follows: presence of opportunistic infections (3 cases), acute probable infectious events (11 cases), invalidated self-reported physical activity questionnaires (21 cases), and invalidated signed informed consent forms (28 cases). Excluded patients per criterion are detailed in Figure A1.

### 2.4. Data Collection

Demographic data (age, sex, living environment, education level, and marital status) and medical history regarding comorbidities (hypertension, diabetes, dyslipidemia, obesity), duration of HIV diagnosis, clinical-immunological stage of infection, number of ART regimens experienced, type, and duration of current therapy were collected from the clinic’s database. Patients were classified based on their clinical-immunological stage into AIDS and non-AIDS groups, according to the Centers for Disease Control and Prevention (CDC) 1993 classification [13].

During a routine monitoring visit, patients underwent a complete clinical examination, including measurement of abdominal circumference, systolic and diastolic blood pressure, and had blood drawn for standard laboratory tests [12]. Therapeutic adherence was systematically assessed by calculating the percentage of antiretroviral (ARV) doses self-reported as taken by the patient during the past 30 days, relative to the number of doses prescribed [14]. The following laboratory blood data were collected: CD4 count, HIV-RNA viral load, leukocyte count, hemoglobin, platelets, blood glucose, total cholesterol, HDL, LDL, and triglycerides. Dyslipidemia was defined as dysregulation in the lipid profile [15]. C-reactive protein (CRP) and interleukin-6 (IL-6) were assessed using validated clinical laboratory assays, as part of routine patient monitoring.

### 2.5. Definition of Metabolic Syndrome

Metabolic syndrome (MetS) was defined by the presence of at least three of the following criteria: obesity, hypertension, elevated triglyceride levels (≥150 mg/dL or treatment for hypertriglyceridemia), low high-density lipoprotein cholesterol (HDL) levels (<40 mg/dL in men and <50 mg/dL in women), and insulin resistance or hyperglycemia (fasting blood glucose ≥100 mg/dL or type 2 diabetes) [7]. Patients were categorized as obese if they had an abdominal circumference greater than 102 cm in men and 88 cm in women [16,17].

### 2.6. Physical Activity Assessment

Physical activity levels were evaluated using the self-reported General Practice Physical Activity Questionnaire (GPPAQ) [18,19]. For this analysis, the standard four GPPAQ categories (Active, Moderately Active, Moderately Inactive, Inactive) were collapsed into three groups: ‘Active’, ‘Moderately Active/Inactive’, and ‘Inactive’ to increase statistical power.

### 2.7. Cardiovascular Risk Assessment

We calculated the 5-year and 10-year cardiovascular risk (CVR) using the Data Collection on Adverse Events of Anti-HIV Drugs (D:A:D^®^) score for PLWH, incorporating traditional risk factors (age, sex, smoking, family history of cardiovascular disease and diabetes, total cholesterol, HDL, and LDL levels) along with CD4 immune status [20]. Patients were stratified into the following risk categories for cardiovascular events: <1%, 1–5%, and >5%. For patients over 40 years, we additionally calculated the 10-year CVR using the SCORE2 algorithm, identifying the corresponding risk age, which was then compared with the chronological age [6,20,21]. Based on the D:A:D^®^ 5-year score, a CVR > 5% was considered significant. To analyze factors associated with this risk, numerical data were grouped into categorical variables: age over 40 years (Yes/No), undetectable HIV-RNA (Yes/No), CD4 > 500/mm^3^ (Yes/No), dyslipidemia (Yes/No), AC (normal/high), CRP > 5 mg/dL (Yes/No), IL-6 > 10 pg/mL (Yes/No) [16].

### 2.8. Laboratory Procedures

Blood samples were collected in the morning after an overnight fast by trained medical staff, following standard venipuncture procedures. Laboratory tests were performed according to local protocols aligned with the European AIDS Clinical Society (EACS) guidelines [12].

### 2.9. Statistical Analysis

For the statistical analysis, we used XLSTAT statistical analysis software, version 2020.1. Descriptive statistics were used to summarize categorical variables as absolute and relative frequencies, while numerical variables were described using mean, standard deviation, median, range, data distribution, and normality testing. Univariate analyses were performed using appropriate statistical tests depending on the variable type: chi-square or Fisher’s exact tests for categorical variables, and Student’s *t*-test or Mann–Whitney U test for continuous variables. Variables associated with significant cardiovascular risk (defined as the 5-year D:A:D^®^ score ≥ 5%) in univariate analysis at a *p*-value < 0.1, along with those deemed clinically relevant based on prior literature, were considered for inclusion in the multivariate logistic regression models. We examined pairwise correlations between covariates (Pearson/Spearman) and additionally performed a principal component analysis (PCA) to detect potential collinearity patterns. Multivariate logistic regression was then used to identify independent predictors, with results reported as odds ratios (ORs) and 95% confidence intervals (CIs) were reported. A two-sided *p*-value < 0.05 was considered statistically significant

## 3. Results

### 3.1. Demographic, Clinical, and Biological Characteristics of PLWH

The study group consisted of 112 subjects aged 38.97 ± 9.95 years, predominantly male (58%). Most participants had at least 12 years of formal education (71%) and lived in urban areas (73%). Only 24% of patients reported an active lifestyle, while 29% consumed alcohol, and 45% were smokers. No significant sex differences were observed in smoking, physical activity, or HIV-related outcomes. However, males more frequently had higher levels of formal education, resided in urban areas, and reported alcohol consumption (Table 1).

Notably, there were 44 PLWH over the age of 40 years and 68 under 40 years. The latter group was divided into two categories based on HIV transmission method: 34 were infected in early childhood and have grown up living with HIV as part of the Romanian Pediatric Cohort, while the other 34 acquired the infection in adulthood, primarily through sexual transmission (non-cohort group) [22].

The duration since HIV diagnosis ranged from 1 to 30 years, with a median of 11 years. The median number of previously experienced antiretroviral regimens was 3, and the median duration of the current ART regimen was 2 years.

The AIDS stage was identified in 60% cases, with a nadir of CD4 below 200/mm^3^ in 54% of patients. As a result of ART, 80% of patients had undetectable HIV-RNA and 60% had CD4 immunity over 500/mm^3^ [Table 1].

Mean hematological parameters were generally within normal reference ranges and did not significantly influence statistical stratification. Nevertheless, sex-related differences were observed, with males exhibiting higher CRP and triglyceride levels. Additionally, elevated inflammatory markers were identified, with CRP >5 mg/dL in 23.2% of patients and IL-6 >10 pg/mL in 28.5% (Table 2).

### 3.2. Metabolic Syndrome in PLWH

Overall, the abdominal circumference ranged from 55 cm to 120 cm. According to sex-specific reference values, 24.11% (27/112) of patients had abdominal obesity, a factor associated with cardiovascular risk. Hypertension was identified in 27.68% (31/112) of patients, dyslipidemia in 72.21% (82/112), and hyperglycemia in 7.07% (7/112) [Table 2].

Around 25% (28/112) of patients met three or more criteria for the diagnosis of MetS. As expected, MetS was more prevalent among PLWH over 40, reaching 41%. Dyslipidemia was the most common component of MetS across all age groups.

**Figure A2:**
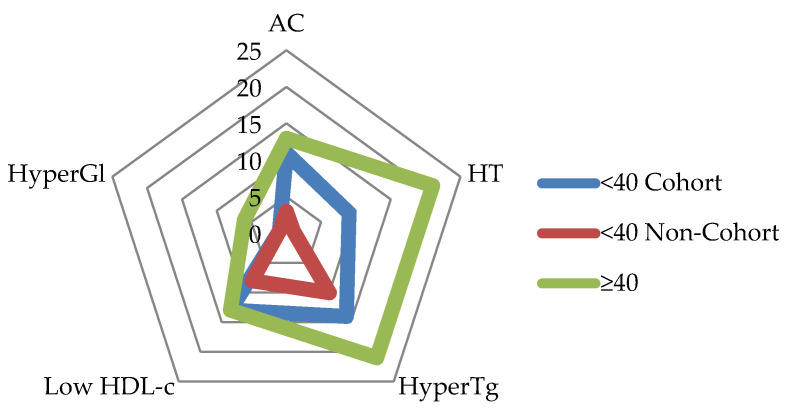
Metabolic syndrome criteria by PLWH group. Legend: <40 Cohort: group of PLWH under age 40, part of the Romanian Pediatric Cohort; <40 Non-Cohort: group of PLWH under age 40, distinct from Romanian Pediatric Cohort; >40: group of PLWH over 40 years old; AC: abdominal circumference; HT: hypertension; HyperTg: hypertriglyceridemia (>150 mg/dL); Low HDL-c: high-density lipoprotein cholesterol (<40 mg/dL in men and <50 mg/dL in women); Hypergl: hyperglycemia (fasting blood glucose ≥ 100 mg/dL or type 2 diabetes).

A higher prevalence of hypertension and obesity was observed in PLWH under 40 years from the pediatric HIV cohort (*N* = 34) in comparison with the non-cohort group, contributing to different MetS rates: 23.5% versus 5.9% (Figure A2).

### 3.3. Cardiovascular Risk in PLWH

The 10-year CVR score estimated using the SCORE2 algorithm in PLWH over the age of 40 years had a mean value of 9.86 ± 4.91%. CVR in this group also revealed a mean cardiovascular age of 7.5 ± 5.10 years higher than the chronological age, suggesting a pattern of accelerated aging in this population [19,20]. The 10-year CVR calculated by the D:A:D^®^ algorithm found a mean value of 10.13 ± 7.45. We observed a very strong correlation between the two methods (correlation coefficient r = 0.95; *p* < 0.001).

**Figure 1:**
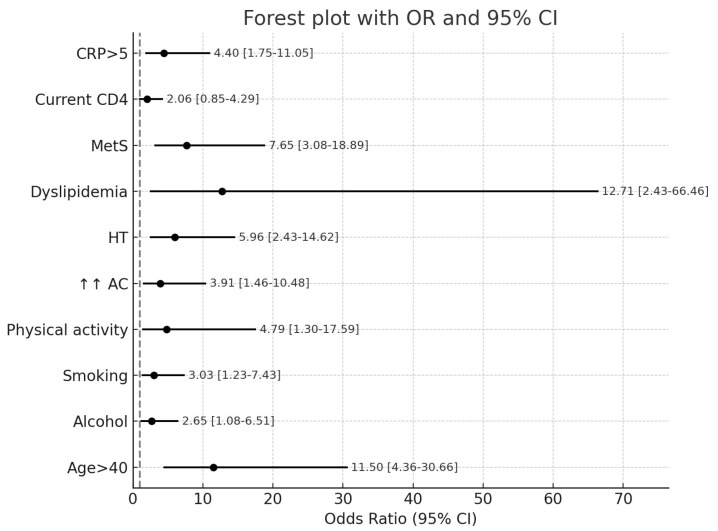
Forest plot of the association of cardiovascular disease risk factors for people living with HIV. Legend: AC: increased abdominal circumference (abdominal obesity); HT: hypertension; CRP: C-reactive protein; MetS: metabolic syndrome.

Overall, using the D:A:D^®^ algorithm, we found that 23.21% of PLWH had a 5-year CVR higher than 5%. In analyzing the influence of demographic, behavioral, metabolic, and HIV-specific factors, we found significant associations between cardiovascular risk and age over 40 years, smoking, alcohol consumption, and insufficient engagement in physical activity. In contrast, education level, living environment, and sex were not significantly associated. HIV-specific factors, including duration of diagnosis, AIDS stage, HIV-RNA levels, CD4 count, duration and ART regimen, did not significantly influence the likelihood of a 5-year cardiovascular risk exceeding 5%. Among inflammatory markers, elevated CRP levels (but not IL-6) were associated with a CVR > 5% (Table 3; Figure 1).

**Figure A3:**
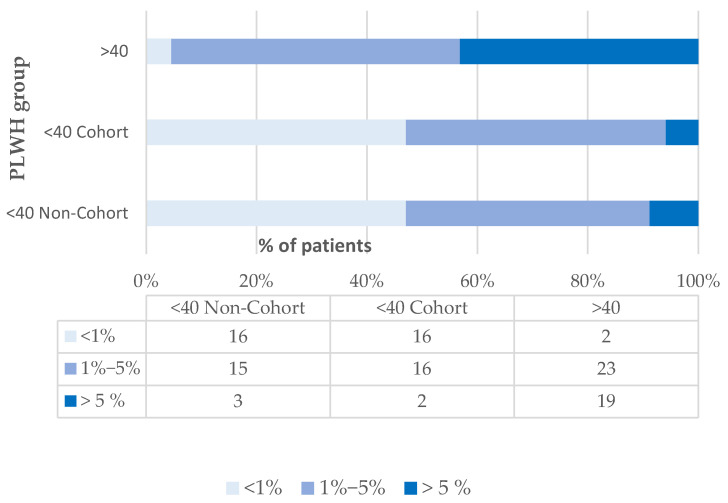
Distribution of cardiovascular risk scores by PLWH group, according to the D:A:D^®^ algorithm. Legend: <40 Cohort: group of PLWH under age 40, part of the Romanian Pediatric HIV Cohort; <40 Non-Cohort: group of PLWH under age 40, distinct from Romanian Pediatric Cohort; >40: group of PLWH over 40 years old.

According to the D:A:D algorithm, the 5-year CVR score for PLWH under the age of 40 years had a mean value of 1.5% ± 1.4, with no significant differences between patients from the pediatric HIV cohort and non-cohort groups (1.6% ± 1.6% vs. 1.4% ± 1.25; *p* = 0.681) (Figure A3).

## 4. Discussion

Our results indicate a strong correlation between the SCORE2 and D:A:D^®^ algorithms for assessing cardiovascular risk in PLWH over 40 years. The findings suggest more advanced “cardiovascular aging” compared to chronological age, further associated with traditional risk factors and elevated CRP levels.

### 4.1. Cardiovascular Risk and Inflammation in Our Study, CVR Correlated with CRP but Not with IL-6

The value of CRP as a biomarker in assessing cardiovascular risk is supported by its inclusion in several prevention guidelines, most recently in the 2024 ESC Guidelines for Chronic Coronary Syndrome [23]. However, its clinical applicability is limited by genetic variability and by the fact that it primarily reflects systemic rather than vascular-specific inflammation. IL-6, in contrast, occupies a more central position in the inflammatory cascade and is a key factor in the pathophysiology of cardiovascular diseases. Produced by macrophages, monocytes, endothelial cells, vascular smooth muscle cells, and fibroblasts, IL-6 plays an important role in the development of atherosclerotic disease progression and plaque destabilization, thereby increasing susceptibility to rupture [24]. Beyond stimulating the synthesis of acute-phase proteins, including C-RP, IL-6 demonstrates superior predictive value for cardiovascular risk [25,26]. Nonetheless, its clinical utility is limited by its short half-life and considerable intraindividual variability, influenced by factors like the postprandial state, physical activity, and circadian variations. At present, no validated tests or standardized protocols exist for sample collection to reliably assess IL-6 levels and their impact on cardiovascular risk. In contrast, CRP has a longer half-life and more stable levels, making it a more practical biomarker for cardiovascular risk assessment [27].

In our cohort, IL-6 levels were likely influenced by the timing of the blood sampling, variations in the wake-up time on the day of collection, commuting distance from home to the hospital and patients’ physical activity level early in the morning, thus not allowing for an accurate estimation. Further studies with strictly controlled blood drawing conditions could compensate for this limitation.

### 4.2. Infection with HIV and the Cardiovascular Risk

Our study found a strong correlation between the standard SCORE2 score (adjusted for country-specific risk) and the D:A:D^®^ score for PLWH, estimated over a 10-year period for individuals over 40 years old. However, standard risk scores are not applicable for individuals under 40, despite the significant CVR in PLWH due to accelerated aging [28].

The updated guidelines of the American Heart Association suggest using locally validated standard risk scores while adjusting the estimated risk by a factor of 1.5 to 2 in PLWH, particularly in cases of persistent viremia or other high-risk markers [23].

Various guidelines for CVR calculations, whether HIV-specific or general, reflect the low predictive accuracy of these models in PLWH, as well as the variability of absolute atherosclerotic disease risk across different regions [28]. Standard CVR prediction scores for the general population tend to underestimate the risk in PLWH, who exhibit two distinct types of myocardial infarction (MI). Plaque rupture and atherothrombosis are the primary mechanisms of MI in the general population, but they account for only 50% of MIs in PLWH. The remaining cases occur in the absence of atherogenesis, driven by structural abnormalities of the coronary vessels, dilated cardiomyopathy, and the influence of other HIV-specific factors [29]. Inflammation and the activation of both innate and adaptive immune responses play a crucial role in atherogenesis and the pathogenesis of cardiovascular diseases in the general population, a process exacerbated by changes associated with HIV infection [30,31].

A prospective study of the Danish HIV cohort, compared to the general population, which assessed subclinical and obstructive coronary atherosclerosis (≥50% stenosis) using coronary computed tomography angiography, demonstrated that HIV is independently associated with a twofold higher risk of any form of subclinical coronary atherosclerosis and a threefold higher risk of obstructive coronary atherosclerosis. These results were reported after adjusting for cardiovascular risk factors, including age, sex, hypertension, dyslipidemia, active smoking, overweight or obesity, and diabetes, providing a possible explanation for the increased risk of myocardial infarction in PLWH [4].

Metabolic syndrome (MetS), a cluster of risk factors including central obesity, hypertension, dyslipidemia, and insulin resistance, is a significant contributor to cardiovascular morbidity. Its prevalence is increasing among PLWH, influenced by both traditional and HIV-related factors, including ART. A meta-analysis of 102 studies from five continents found an overall combined prevalence of MetS in PLWH of 25.3%, with a 1.5-fold higher risk among individuals exposed to ART and a 1.6-fold higher risk than in HIV-uninfected individuals [5].

Antiretroviral therapy (ART) has significantly increased the life expectancy of PLWH. However, despite achieving complete viral suppression under therapy, PLWH maintain a persistently elevated inflammatory state in comparison with the non-HIV population. This is explained by the persistence of HIV in reservoirs, intestinal bacterial translocation, co-infections—particularly with cytomegalovirus–and the incomplete recovery of adaptive immune deficits altered by HIV. Epidemiological studies indicate an increased risk of cardiovascular events or advanced atherosclerosis in PLWH with elevated inflammatory biomarkers, increased monocyte activation, and a prothrombotic state (e.g., elevated D-dimer levels) [28,32]. While older-generation antiretroviral drugs had been associated with an increased risk of myocardial infarction, newer agents exert a lesser impact on lipid profiles, suggesting a potentially lower cardiovascular risk [33]. Overall, the benefits of early ART initiation are unquestionable, as viral suppression is associated with a lower risk of opportunistic infections and cardiovascular complications, along with reduced residual inflammatory levels in comparison with those who start ART late due to delayed diagnosis [34,35].

### 4.3. Perspective of Cardiovascular Risk in Pediatric HIV Cohorts

Our cohort includes a distinct subgroup of young adults who acquired HIV in early childhood (1988–1990) due to nosocomial transmission. They are part of Romania’s distinctive pediatric HIV cohort, the largest in Europe [22]. These individuals have been living with chronic HIV infection since infancy, often since their first year of life, raising specific concerns about the long-term impact of viral persistence on neuroendocrine and cardiovascular development. To our knowledge, no studies have specifically addressed CVR in pediatric populations with postnatally acquired HIV, comparable to our cohort.

Findings from studies on perinatally HIV-infected cohorts report cardiometabolic dysfunctions distinct from those observed in adult PLWH. The determinants of these dysfunctions are multifactorial, and are influenced by lifestyle, genetics, individual and social characteristics. Prolonged exposure to HIV, as well as to ART, starting from infancy, may contribute to the premature onset of cardiometabolic comorbidities [36].

Our results demonstrated a greater prevalence of MetS among individuals in the HIV cohort in comparison with the non-cohort HIV-infected young adults, although the CVR remained similarly low. Growing up with HIV, along with the experiences of young adulthood in terms of traditional health risk factors, cumulative comorbidities, and environmental exposures, represents a unique condition that may additionally contribute to a higher cardiovascular risk [34]. Although this cohort is nationally specific, similar patient populations may exist in other regions with a history of pediatric HIV epidemics. Thus, our findings may provide relevant insights for clinicians managing long-term survivors of perinatally or early childhood-acquired HIV, particularly regarding early-onset metabolic and cardiovascular risks and the need for intensified screening and prevention strategies in this vulnerable subgroup.

### 4.4. Strategies and Interventions for Preventing Cardiovascular Events

Our study demonstrated the major contribution of traditional risk factors and the role of inflammation in CVR, as evaluated by the 5-year DAD^®^ algorithm. However, it did not reveal the impact of HIV-specific factors such as the stage of immunodeficiency, viral load, adherence, or ART history. This observation may be attributable to the stable clinical status of the study participants, who largely demonstrated optimal therapeutic adherence and sustained viral suppression, without significant chronic comorbidities or co-infections. Smoking and alcohol consumption were the primary high-risk behaviors, while no illicit drug use was reported. In line with these findings, the main objective for the clinical management of CVR in PLWH at our center is to promote a healthy lifestyle. This includes regular physical activity, a balanced diet, maintaining an optimal body weight, and modifying high-risk behaviors. ART adherence and achieving sustained viral suppression should remain key priorities to limit immune activation and systemic inflammation associated with HIV and CVR [26,28,37].

New data are promising regarding the utility of the C-reactive protein–triglyceride–glucose index as a novel biomarker for insulin resistance and inflammation, as well as its association with stroke risk. However, data on people living with HIV (PLWH) are lacking, and future research should address this population [38].

Incident atrial fibrillation was significantly associated with HIV severity, with few distinctive electrophysiological characteristics. The increased mid- and long-term rates of arrhythmia recurrence and atrial fibrillation observed in this population underscore the need for vigilant monitoring and timely referral for catheter ablation in our clinical practice [39].

The treatment of hypertension and dyslipidemia requires close collaboration with cardiologists. Current cardiovascular disease treatment guidelines do not consider HIV status, and there is a lack of data on specific criteria for initiating various preventive CV interventions in PLWH [40]. The REPRIVE study demonstrated that pitavastatin use in PLWH exerts anti-inflammatory effects and reduces CVR, subsequently establishing its role as a standard of care [41]. Other anti-inflammatory interventions currently under investigation in PLWH target microbial translocation and chronic co-infections, particularly cytomegalovirus, which is considered a contributing factor to coronary artery disease and reactivation of HIV from latently infected cells. Achieving a functional HIV cure could help reduce immune activation, thus providing cardiovascular benefits. However, the effectiveness of anti-inflammatory strategies based on immunosuppressive therapies remains uncertain, as most clinical trials do not include PLWH [32,42].

### 4.5. Limitations of the Study

The present study has several important limitations that must be acknowledged. Because this study included all available HIV individuals under care at our center, presented in a time-specific interval, no a priori sample size calculation was performed. We acknowledge that the relatively small sample size represents a limitation of the study in terms of statistical power and generalizability of the results. We acknowledge the potential for selection bias due to scheduling, and we compare demographic and clinical characteristics between the patients scheduled for visits in April and October, with no significant differences. This suggests the study sample is representative of the larger clinic population. The study cohort, while clinically diverse, is inherently heterogeneous in terms of transmission routes, duration of infection, age of diagnostic ART regimens and treatment duration. This variability complicates efforts to draw unified conclusions about cardiovascular risk across the population. Contextual limitations should be considered in the interpretation of the findings of this study. The analysis was conducted in a single HIV clinic in southeastern Romania, which may affect the generalizability of the results to broader populations of PLWH in different geographic or healthcare settings.

Another limitation is the use of the self-reported General Practice Physical Activity Questionnaire to assess physical activity levels. While this instrument can provide a valuable insight into the usual individual exercise level, it holds the bias of erroneous recall, which can further lead to misclassification.

Given the numerous factors that influence CVR, a larger sample size would be essential for drawing robust and generalizable conclusions. Nevertheless, we believe that our findings should be interpreted as preliminary and exploratory, with the primary value of the study lying in hypothesis generation rather than direct clinical application. Further research, ideally involving multicenter and longitudinal designs, is needed to validate and expand upon these initial observations.

## 5. Conclusions

This study highlights the importance of the early assessment of MetS and CVR in PLWH, including younger and clinically stable populations. Residual inflammation in PLWH, even in patients with full viral suppression under ART, is associated with increased CVR. Routine integration of metabolic risk screening into HIV care may support timely prevention and personalized management strategies. While the limited sample size and single-center setting constrain the generalizability of our findings, the observed trends may be relevant for clinicians managing long-term survivors of childhood-acquired HIV. These preliminary findings underscore the need for continued cardiovascular monitoring in PLWH. We hope our results will aid in the design of future, large-scale, multicenter studies focused on this particular population.

## Figures and Tables

**Table 1:** Characteristics of PLWH stratified by demographic data, behavioral habits, and AIDS-related outcomes.

	Categories	Overall (112)		Female (48)		Male (64)		χ^2^ Test *p*
		n	%	n1	%	n2	%	
Age	≥40 years old	44	39%	17	35%	27	43%	0.380
	<40 years old	68	61%	33	65%	36	57%	
Living	Rural	30	27%	20	41%	10	16%	0.003
	Urban	82	73%	29	59%	53	85%	
High school education/over	No	33	29%	29	41%	50	79%	0.020
	Yes	79	71%	20	59%	13	21%	
Smoking	Yes	50	45%	19	39%	31	49%	0.270
	No	62	55%	30	61%	32	51%	
Alcohol	Yes	33	29%	5	10%	28	44%	<0.001
	No	79	71%	44	90%	35	56%	
Physical activity index	Inactive	22	21%	12	25%	10	25%	0.522
	Moderate	63	56%	26	53%	37	59%	
	Active	27	24%	11	22%	16	16%	
AIDS	Yes	67	60%	30	61%	37	59%	0.789
	No	45	40%	19	39%	26	41%	
Current CD4	<500/mm^3^	44	39%	18	37%	27	43%	0.512
	≥500/mm^3^	68	61%	31	63%	36	57%	
Current ARN-HIV	Detectable	22	20%	13	26%	9	14%	0.105
	Undetectable	90	80%	36	74%	54	86%	
ARV Adherence	≥95%	70	63%	29	59%	41	65%	0.522
	<95%	41	37%	20	41%	22	35%	
Current ARV	BIC	49	44%	16	33%	32	51%	0.127
	DLG	25	22%	12	25%	12	19%	
	DOR	15	13%	10	20%	5	8%	
	Others	13	12%	11	22%	14	22%	

Legend: ARV: antiretrovirals; BIC: bictegravir; DLG-NRTI: dolutegravir-based regimens; DEL: doravirine/lamivudine/tenofovir; CD4: cluster of differentiation 4 co-receptor for the T-cell receptor; HIV-RNA: quantitative test of HIV ribonucleic acid with a detecting limit of 40 copies/mL.

**Table 2:** Biological profile of people living with HIV by sex.

	Average ± SD			Two-Sample *t*-Tests
	Overall	Female	Male	
WBCs [_/mm^3^]	6280 ± 2049	5932 ± 2097	6550 ± 1986	0.116
Platelets [_/mm^3^]	231,926 ± 69,563	240,796 ± 68,559	225,029 ± 70,099	0.234
CRP [mg/dL] *	4.85 ± 10.82	2.80 ± 3.17	6.45 ± 14.04	0.048
IL-6 [pg/mL] **	7.66 ± 6.09	7.17 ± 5.20	8.03 ± 6.72	0.445
Glycemia [mg/dL]	104.24 ± 16.09	101.90 ± 12.65	106.06 ± 18.22	0.157
Cholesterol total [mg/dL]	211.83 ± 45.83	214.83 ± 45.08	209.55 ± 46.64	0.546
HDL cholesterol [mg/dL]	55.68 ± 23.18	59.53 ± 18.07	54.47 ± 26.42	0.232
LDL cholesterol [mg/dL]	122.42 ± 40.92	121.04 ± 40.47	123.20 ± 41.57	0.818
Triglycerides [mg/dL]	150.51 ± 97.99	127.28 ± 62.20	168.587 ± 115.92	0.017

Legend: * CRP: C-reactive protein normal values < 0.5 mg/dL ng/mL; ** IL-6: interleukin 6 with normal values < 9.7 pg/mL; WBCs: white blood cells; Hb: hemoglobin.

**Table 3:** Comparative analysis of 5-year CVR according to DAD^®^ algorithm by traditional factors and AIDS-related risk factors.

			CVR ≥ 5% n1 = 26 23.21%	CVR < 5% n2 = 86 76.78%	OR	CI: 95	*p* (χ^2^)
Demographic factors	Age	>40 years	21	23	11.50	4.36; 30.66	<0.001
		<40 years	5	63			
	Sex	Male	18	45	2.05	0.81; 5.16	0.104
		Female	8	41			
	Formal education	≥12 years	15	64	2.13	0.86; 5.27	0.101
		<12 years	11	22			
	Living	Urban	20	62	1.29	0.46; 3.596	0.626
		Rural	6	24			
Behavioral factors	Alcohol	Yes	12	21	2.65	1.08; 6.51	0.033
		No	14	65			
	Physical activity	Inactive	10	12	4.79	1.30; 17.59	0.018
		Active	4	23			
	Smoking	Yes	17	33	3.03	1.23; 7.43	0.015
		No	9	56			
Metabolic syndrome	Obesity	Yes	9	14	3.10	1.12; 8.57	0.028
		No	12	58			
	HT	Yes	15	16	5.96	2.43; 14.62	<0.001
		No	11	70			
	Dyslipidemia	Yes	25	57	12.71	2.43; 66.46	0.002
		No	1	29			
	Metabolic syndrome	Yes	15	13	7.65	3.08; 18.98	<0.001
		No	11	73			
HIV-related factors	AIDS	Yes	16	51	1.09	0.44; 2.69	0.838
		No	10	35			
	Duration of HIV diagnostic	>5 years	20	62	1.29	0.46; 3.59	0.626
		<5 years	6	24			
	Previous ART	>3	7	37	0.487	0.18; 1.26	0.140
		≤3	19	49			
	Current CD4	>500/mm^3^	12	55	2.06	0.85; 4.98	0.104
		<500/mm^3^	14	31			
	RNA-HIV	detectable	4	18	1.45	0.44; 4.73	0.532
		undetectable	22	68			
	CRP	≥5 mg/L	12	14	4.40	1.75; 11.05	0.001
		<5 mg/L	14	72			
	IL6	>10 pg/ml	7	25	1.11	0.41; 2.97	0.831
		<10 pg/ml	19	61			

Legend: AIDS: acquired immunodeficiency syndrome; ART: antiretroviral treatment regimens; CRP: C-reactive protein; HT: hypertension; IL6: interleukin 6; MetS: metabolic syndrome.

## Data Availability

The data presented in this study are available and can be shared upon reasonable request sent to the corresponding authors.

## Abbreviations

AC	Abdominal circumference
AIDS	Acquired immunodeficiency syndrome
ART	Antiretroviral therapy
Av	Average
BIC	Bictegravir
CD4	Cluster of differentiation 4 co-receptor for the T-cell receptor
CDC	Centers for Disease Control and Prevention
CRP	C-reactive protein
CVR	Cardiovascular risk
D:A:D^®^	Data Collection on Adverse Events of Anti-HIV Drugs Study
EACS	European AIDS Clinical Society
EAPC	European Association of Preventive Cardiology
DOR	Doravirine
DLG	Dolutegravir
GPPAQ	General Practice Physical Activity Questionnaire
HT	Hypertension
HDL	High-density lipoprotein cholesterol
HIV	Human immunodeficiency virus
HIV-RNA	Test of HIV ribonucleic acid
IL-6	Interleukin-6
LDL	Low-density lipoprotein cholesterol
MetS	Metabolic syndrome
NRTI	Nucleoside reverse transcriptase inhibitors
OR	Odd ratio
PLWH	People living with HIV/AIDS
SCORE2	Systematic Coronary Risk Evaluation 2
SD	Standard deviation
UNAIDS	Joint United Nations Programme on HIV/AIDS
WBC	White blood cell
WHO	World Health Organization

## References

[1] UNAIDS DATA 2024 (2024). Geneva: Joint United Nations Programme on HIV/AIDS. https://www.unaids.org/sites/default/files/media_asset/UNAIDS_FactSheet_en.pdf.

[2] Trickey A., McGinnis K., Gill M.J., Abgrall S., Berenguer J., Wyen C., Hessamfar M., Reiss P., Kusejko K., Silverberg M.J. (2024). Longitudinal trends in causes of death among adults with HIV on antiretroviral therapy in Europe and North America from 1996 to 2020: A collaboration of cohort studies. Lancet HIV.

[3] Nomah D.K., Jamarkattel S., Bruguera A., Moreno-Fornés S., Díaz Y., Alonso L., Aceitón J., Llibre J.M., Domingo P., Saumoy M. (2024). Evolving AIDS- and non-AIDS Mortality and Predictors in the PISCIS Cohort of People Living With HIV in Catalonia and the Balearic Islands (Spain), 1998–2020. Open Forum Infect. Dis..

[4] Knudsen A.D., Fuchs A., Benfield T., Køber l., Nordestgaard B.G., Afzal S., Kuhl J.T., Sigvardsen P.F., Suarez-Zdunek M.A., Gelpi M. (2025). HIV Is Associated With Subclinical Coronary Atherosclerosis: A Prospective Matched Cohort Study. Clin. Infect. Dis..

[5] Trachunthong D., Tipayamongkholgul M., Chumseng S., Darasawang W., Bundhamcharoen K. (2024). Burden of metabolic syndrome in the global adult HIV-infected population: A systematic review and meta-analysis. BMC Public Health.

[6] SCORE2 working group and ESC Cardiovascular risk collaboration (2021). SCORE2 risk prediction algorithms: New models to estimate 10-year risk of cardiovascular disease in Europe. Eur. Heart J..

[7] World Obesity Federation (2024). World Obesity Atlas 2024. London: World Obesity Federation. https://data.worldobesity.org/publications/?cat=22.

[8] Pantazis N., Porter K., Sabin C.A., Burns F., Touloumi G. (2024). Antiretrovirals and obesity. Lancet HIV.

[9] Chandiwana N., Manne-Goehler J., Gaayeb L., Calmy A., Venter W.D.F. (2024). Novel anti-obesity drugs for people with HIV. Lancet HIV.

[10] Manne-Goehler J., Siedner M.J. (2024). Untangling the causal ties between antiretrovirals and obesity. Lancet HIV.

[11] Compartimentul Pentru Monitorizarea si Evaluarea Infectiei HIV/SIDA in Romania. Institutul National de Boli Infectioase “Prof. Dr. Matei Bals” Date Statistice: 1 Decembrie Evoluția Fenomenului HIV în România 2023–2024. https://www.cnlas.ro/index.php/date-statistice.

[12] European AIDS Clinical Society (EACS) Guidelines 2023, VS.12.0. https://www.eacsociety.org/media/guidelines-12.0.pdf.

[13] Kamps B.S., Brodt H.R., Staszewski S., Bergmann L., Helm E.B. (1994). AIDS-free survival and overall survival in HIV infection: The new CDC classification system (1993) for HIV disease and AIDS. Clin. Investig..

[14] Yeneakal K.A., Teferi G.H., Mihret T.T., Mengistu A.K., Tizie S.B., Tadele M.M. (2025). Predicting antiretroviral therapy adherence status of adult HIV-positive patients using machine learning: Northwest Ethiopia, 2025. BMC Med. Inform. Decis. Mak..

[15] Gaita L., Timar B., Timar R., Fras Z., Gaita D., Banach M. (2024). Lipid Disorders Management Strategies (2024) in Prediabetic and Diabetic Patients. Pharmaceuticals.

[16] WHO European Regional Obesity Report 2022 (2022). Copenhagen: WHO Regional Office for Europe. https://iris.who.int/bitstream/handle/10665/353747/9789289057738-eng.pdf.

[17] Institutul Național de Sănătate Publică (2023). Ghid de Prevenție Pentru Medicul de Familie. Intervenții Preventive Integrate Adresate stilului de Viață—Alimentația. Activitatea Fizică. București. https://proiect-pdp1.insp.gov.ro/wp-content/uploads/2023/06/Ghidul-Alimentatia-Activitatea-fizica.pdf.

[18] Golightly Y.M., Allen K.D., Ambrose K.R., Stiller J.L., Evenson K.R., Voisin C., Hootman J.M., Callahan L.F. (2017). Physical Activity as a Vital Sign: A Systematic Review. Prev. Chronic Dis..

[19] Ahmad S., Harris T., Limb E., Kerry S., Victor C., Ekelund U., Iliffe S., Whincup P., Beighton C., Ussher M. (2015). Evaluation of reliability and validity of the General Practice Physical Activity Questionnaire (GPPAQ) in 60–74 year old primary care patients. BMC Fam. Pract..

[20] Centre of Excelence for Health, Immunity and Infections Clinical Risk Scores. https://chip.dk/Resources/Clinical-risk-scores.

[21] European Association of Preventive Cardiology Heart Score. Calculate 10-Year Risk of Fatal and Non-Fatal Cardiovascular Disease Events. https://www.heartscore.org/en_GB?_gl=1*snk2xq*_gcl_au*MTg0MjAxOTYyMy4xNzQwNzQ2NjA4*_ga*NTg0NzgwNjU0LjE3NDA3NDY2MzE.*_ga_5Y189L6T14*MTc0MjExNzM2MS45LjAuMTc0MjExNzM2MS42MC4wLjA.*_ga_VPF4X3T28K*MTc0MjExNzM2MS41LjAuMTc0MjExNzM2MS4wLjAuMA.

[22] Preda M., Manolescu L.C.S. (2022). Romania, a Harbour of HIV-1 Subtype F1: Where Are We after 33 Years of HIV-1 Infection?. Viruses.

[23] Vrints C., Andreotti F., Koskinas K.C., Rossello X., Adamo M., Ainslie J., Banning A.P., Budaj A., Buechel R.R., Chiariello G.A. (2024). 2024 ESC Guidelines for the management of chronic coronary syndromes. Eur. Heart J..

[24] Zhang H., Dhalla N.S. (2024). The Role of Pro-Inflammatory Cytokines in the Pathogenesis of Cardiovascular Disease. Int. J. Mol. Sci..

[25] Lin G.M., Lloyd-Jones D.M., Colangelo L.A., Lima J.A.C., Szklo M., Liu K. (2024). Association between secondhand smoke exposure and incident heart failure: The Multi-Ethnic Study of Atherosclerosis (MESA). Eur. J. Heart Fail..

[26] Mehta N.N., de Goma E., Shapiro M.D. (2024). IL-6 and Cardiovascular Risk: A Narrative Review. Curr. Atheroscler. Rep..

[27] Tükenmez Tigen E., Gökengin D., Özkan Özdemir H., Akalın H., Kaya B., Deveci A., İnan A., İnan D., Altunsoy A., Özel A.S. (2024). Prevalence of Cardiovascular Disease and Comparison of Risk Category Predictions of Systemic Coronary Risk Evaluation Score-2 and 4 Other Cardiovascular Disease Risk Assessment Tools Among People Living with Human Immunodefficiency Virus in Türkiye. Anatol. J. Cardiol..

[28] Ntsekhe M., Baker J.V. (2023). Cardiovascular Disease Among Persons Living With HIV: New Insights Into Pathogenesis and Clinical Manifestations in a Global Context. Circulation.

[29] Crane H.M., Nance R.M., Avoundjian T., Harding B.N., Whitney B.M., Chow F.C., Becker K.J., Marra C.M., Zunt J.R., Ho E.L. (2021). Types of Stroke Among People Living With HIV in the United States. J. Acquir. Immune Defic. Syndr..

[30] Triant V.A., Lyass A., Hurley L.B., Borowsky L.H., Ehrbar R.Q., He W., Cheng D., Lo J., Klein D.B., Meigs J.B. (2024). Cardiovascular Risk Estimation Is Suboptimal in People With HIV. J. Am. Heart Assoc..

[31] Arbune M. (2021). Premature aging and cardiovascular diseases related to HIV infection. Rom. Arch. Microbiol. Immunol..

[32] Obare L.M., Temu T., Mallal S.A., Wanjalla C.N. (2024). Inflammation in HIV and Its Impact on Atherosclerotic Cardiovascular Disease. Circ. Res..

[33] Grinspoon S.K., Watanabe M., Ribaudo H.J., Bloomfield G.S., Fichtenbaum C.J., Umbleja T., Chu S.M., Fitch K.V., Diggs M.R., Zhao S. (2025). Factors affecting risk of cardiovascular disease (CVD) events in a global CVD prevention cohort of people with human immunodeficiency virus (HIV). Clin. Infect. Dis..

[34] Savinelli S., Newman E., Mallon P.W.G. (2024). Metabolic Complications Associated with Use of Integrase Strand Transfer Inhibitors (InSTI) for the Treatment of HIV-1 Infection: Focus on Weight Changes, Lipids, Glucose and Bone Metabolism. Curr. HIV/AIDS Rep..

[35] Corti N., Menzaghi B., Orofino G., Guastavigna M., Lagi F., Di Biagio A., Taramasso L., De Socio G.V., Molteni C., Madeddu G. (2024). Risk of Cardiovascular Events in People with HIV (PWH) Treated with Integrase Strand-Transfer Inhibitors: The Debate Is Not Over; Results of the SCOLTA Study. Viruses.

[36] López E., Sainz T., Dirajlal-Fargo S., Jao J., Pinto J., Buchanan A.M., McKenna M., Milinkovic A., Puga A.M. (2025). Cardiometabolic Health Burden in Pediatric HIV: Unmet Need in the Contemporary Antiretroviral Therapy Era. Cureus.

[37] Șoldea S., Iovănescu M., Berceanu M., Mirea O., Raicea V., Beznă M.C., Rocșoreanu A., Donoiu I. (2025). Cardiovascular Disease in HIV Patients: A Comprehensive Review of Current Knowledge and Clinical Implications. Int. J. Mol. Sci..

[38] Huo G., Tang Y., Liu Z., Cao J., Yao Z., Zhou D. (2025). Association between C-reactive protein–triglyceride glucose index and stroke risk in different glycemic status: Insights from the China Health and Retirement Longitudinal Study (CHARLS). Cardiovasc. Diabetol..

[39] La Fazia V.M., Pierucci N., Mohanty S., Gianni C., Della Rocca D.G., Compagnucci P., MacDonald B., Mayedo A., Torlapati P.G., Bassiouny M. (2023). Catheter ablation approach and outcome in HIV+ patients with recurrent atrial fibrillation. J. Cardiovasc. Electrophysiol..

[40] Grinspoon S.K., Zanni M.V., Triant V.A., Kantor A., Umbleja T., Diggs M.R., Chu S.M., Fitch K.V., Currier J.S., Bloomfield G.S. (2025). Performance of the pooled cohort equations and D:A:D risk scores among individuals with HIV in a global cardiovascular disease prevention trial: A cohort study leveraging data from REPRIEVE. Lancet HIV.

[41] Grinspoon S.K., Fitch K.V., Zanni M.V., Fichtenbaum C.J., Umbleja T., Aberg J.A., Overton E.T., Malvestutto C.D., Bloomfield G.S., Currier J.S. (2023). Pitavastatin to Prevent Cardiovascular Disease in HIV Infection. N. Engl. J. Med..

[42] Mazzuti L., Turriziani O., Mezzaroma I. (2023). The Many Faces of Immune Activation in HIV-1 Infection: A Multifactorial Interconnection. Biomedicines.

